# Real-Time Edge Neuromorphic Tasting From Chemical Microsensor Arrays

**DOI:** 10.3389/fnins.2021.771480

**Published:** 2021-12-09

**Authors:** Nicholas LeBow, Bodo Rueckauer, Pengfei Sun, Meritxell Rovira, Cecilia Jiménez-Jorquera, Shih-Chii Liu, Josep Maria Margarit-Taulé

**Affiliations:** ^1^Institute of Neuroinformatics, University of Zurich and ETH Zurich, Zurich, Switzerland; ^2^Donders Centre for Cognition, Radboud University, Nijmegen, Netherlands; ^3^Institute of Microelectronics of Barcelona (IMB-CNM), CSIC, Barcelona, Spain

**Keywords:** deep convolutional neural networks, spiking neural networks (SNNs), electrochemical sensors, sensor fusion, neuromorphic engineering, electronic tongue (E-Tongue)

## Abstract

Liquid analysis is key to track conformity with the strict process quality standards of sectors like food, beverage, and chemical manufacturing. In order to analyse product qualities online and at the very point of interest, automated monitoring systems must satisfy strong requirements in terms of miniaturization, energy autonomy, and real time operation. Toward this goal, we present the first implementation of artificial taste running on neuromorphic hardware for continuous edge monitoring applications. We used a solid-state electrochemical microsensor array to acquire multivariate, time-varying chemical measurements, employed temporal filtering to enhance sensor readout dynamics, and deployed a rate-based, deep convolutional spiking neural network to efficiently fuse the electrochemical sensor data. To evaluate performance we created MicroBeTa (*Micro*sensor *Be*verage *Ta*sting), a new dataset for beverage classification incorporating 7 h of temporal recordings performed over 3 days, including sensor drifts and sensor replacements. Our implementation of artificial taste is 15× more energy efficient on inference tasks than similar convolutional architectures running on other commercial, low power edge-AI inference devices, achieving over 178× lower latencies than the sampling period of the sensor readout, and high accuracy (97%) on a single Intel Loihi neuromorphic research processor included in a USB stick form factor.

## 1. Introduction

Liquid analysis systems that assess process quality in sectors like food, beverage, and chemical manufacturing are in rising demand. Driven by increasingly strict regulations, and by the need to boost productivity and to reduce costs, industry has promoted the development of automated systems for monitoring physicochemical properties of products in their manufacturing cycle. To allow process control when and wherever required, such systems must be small, energetically autonomous, and able to operate in real time.

In this context, the use of chemical multisensor arrays as “electronic tongues” stands out due to their capability of recognizing quantitative and qualitative composition of complex solutions. Inspired by human taste, artificial tongues use an array of chemical sensors (i.e., the artificial taste cells) selective—but not specific—to different solution properties. The multivariate sensor responses are then read out in the electrical domain and modeled by appropriate Machine Learning methods.

To manufacture the arrays, microsensors fabricated in semiconductor technologies present advantages such as miniaturization, robustness, high reproducibility, mass fabrication, and ease of integration with readout electronic circuitry, making them particularly suitable for on-site measurements. Fusion algorithms can then be applied to multisensor readouts in order to automatize the analyses. By exploiting the extended coverage of the sensor array, sensor fusion allows to increase the amount of relevant chemical information inferred in the system. Embedded implementations of these algorithms are, nonetheless, still incipient: most of them are constrained by linear modeling, manual definition, or predefined measurement durations.

In the state of the art, taste inference is delayed until finishing voltammetric cycles or transient measurements when recording from the sensor array. To facilitate pattern recognition and prevent overfitting, input data is often mapped into a lower-dimensional space using methods such as principal component analysis (PCA) (Li et al., 2015; Gutiérrez-Capitán et al., 2019) or partial least squares (PLS) (Qiu et al., 2014; Giménez-Gómez et al., 2016; Gutiérrez-Capitán et al., 2019). Subsequent steps then use linear and non-linear algorithms including discriminant analysis (DA) (Escriche et al., 2012), hierarchical cluster analysis (HCA) (Kundu and Kundu, 2013), and support vector machines (SVMs) (Domínguez et al., 2014) for qualitative or quantitative evaluation.

Deep neural networks (DNNs) offer flexible and scalable representations to suit the complexity of representing dynamic data with one single model. In particular, convolutional neural networks (CNNs), are well suited to fuse data from a large number of sensor channels simultaneously—and to learn useful classification functions on this information—while using fewer, shared connection weights than other neural network architectures. DNN implementations running on conventional digital hardware exhibit, however, strong computational requirements that limit their incorporation in mobile and/or compact analytical devices. Spiking neural networks (SNNs) can offer a significant advantage in power efficiency over continuous-valued architectures when implemented on appropriate hardware (Esser et al., 2016). While they are poorly served by conventional von Neumann processors due to both their highly parallel nature and the asynchronous character of sparse spiking sequences, SNNs can attain high energy efficiency on neuromorphic hardware such as IBM's TrueNorth or Intel's Loihi (Akopyan et al., 2015; Davies et al., 2018).

In this study, we pre-trained continuous-valued CNNs and converted them to SNNs following the rate-based approach developed by Rueckauer et al. (2017). This framework allows direct mapping of deep neural network structures, offering accuracies equivalent to non-spiking Artificial Neural Networks (ANNs) and sparse event-driven computation alike to the aforementioned neuromorphic processors. Once converted, computational efficiency can more easily be optimized: zero activations are natively skipped in the activity-driven operation of spiking networks, and accuracy can be tailored to a given latency and power budget in terms of number of additive operations.

In Margarit-Taulé et al. (2019) we demonstrated a preliminary implementation of a portable electronic tongue analyzing temporal microsensor data via PLS-DA and SVMs. This work builds on these results to introduce SNNs as accurate and power efficient models to perform chemometric data fusion on the edge for liquid analysis. To that end, we:

- present the first spiking, near-sensor implementation of taste running on neuromorphic hardware via deep learning models;
- introduce MicroBeTa, a new dataset with temporal readings from a chemical microsensor array acquired in commercial beverages exemplifying industrial solutions. MicroBeTa was created for training and testing the classification performance of machine learning classifiers, and can be used to assess other future neuromorphic implementations for this task;
- propose a small spiking CNN that achieved high accuracy on the MicroBeTa dataset, and that fits on a single Intel neuromorphic research processor;
- compare the performance of the spiking CNN against that of a k-nearest neighbors (k-NN) classifier, a simple method that was recently deployed on the Loihi;
- determine the contribution of each sensor to beverage classification via the random forest algorithm, and demonstrate the relevance of sensor fusion for different combinations of the sensors processed by the algorithms;
- benchmark the neuromorphic implementation against two commercial inference devices—a GPU and a Neural Compute Stick—and validate the implementation in terms of accuracy, energy efficiency, and inference time when processing the dynamic sensor recordings of the MicroBeTa dataset.

These contributions are discussed in detail below.

## 2. Materials and Methods

### 2.1. Electronic Tongue Setup

**Figure 1:**
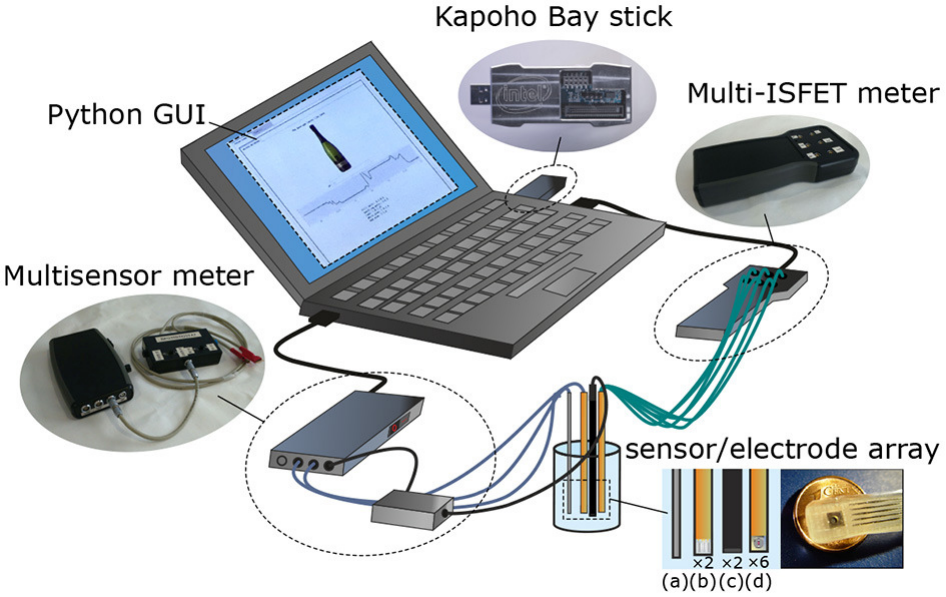
Major components of the neuromorphic electronic tongue used in this work. Detail of sensors and electrodes: 1× temperature Pt-100 (a), 2× Pt microelectrodes (ORP and conductivity, b), 2× reference electrodes (c), 6× ISFET microsensors (d). Adapted from Margarit-Taulé et al. (2019).

The electronic tongue described in this work aims to combine two core hardware components—solid-state electrochemical microsensors and a neuromorphic processor—in a novel system for electrochemical inference. The use of machine learning algorithms facilitates fusion of multivariate sensor readings and modeling of structure within the data while exploiting cross-sensitivity between individual sensors to increase classification accuracy. Figure 1 illustrates the hardware configuration used to acquire the electrochemical readings and to discriminate between beverages. The system employs a chemical sensor array immersed in the beverage under test, two USB readout boards (Giménez-Gómez et al., 2015, 2016), and Intel's Kapoho Bay (KB)—a mobile, USB form factor of the Loihi SNN accelerator —locally attached to a laptop together with the readout boards.

#### 2.1.1. Chemical Sensors and Readout

The sensor array shown in Figure 1 comprises one sensor each for electrical conductivity, temperature, oxidation-reduction potential (ORP), and six ion-sensitive field-effect transistor (ISFET) sensors selective to specific ions (H^+^, Na^+^, K^+^, Ca^2+^, Cl^−^, and ${\text{NO}}_{3}^{-}$). The silicon-based electrochemical sensors were monolithically integrated at the clean room of the Institute of Microelectronics of Barcelona (IMB-CNM). Their technical specifications are given in Table 1. We combined electrochemical measurement techniques of different nature (conductometry, ion-selective potentiometry, reducing or oxidizing (redox) potentiometry) in a hybrid electronic tongue. Merging stable— but general—sensors like conductivity and ORP with more specific ones—but also more unreliable—like the ISFETs allows one to achieve a certain degree of operative independence with respect to unexpected ISFET failure or drift. This approach has already been reported in the literature (Giménez-Gómez et al., 2016; Gutiérrez-Capitán et al., 2019; Margarit-Taulé et al., 2019) as successfully improving the performance of electrochemical microsensor technologies in the chemometric analysis of water and wine.

**Table 1:** Technical specifications of the IMB-CNM's electrochemical microsensors used in this work.

**Parameter**	**Sensor**	**Sensitivity**	**Range**	**Channel label**
Conductivity	Pt μelectrode	900 mV·cm/mS	0.35–12 mS/cm	COND
Redox potential	Pt μelectrode	–	160–650 mV	ORP
pH	ISFET	54 mV/dec	pH 2–13	ISF1
K^+^	ISFET	52 mV/dec	1·10^−5^–1·10^−2^ M	ISF2
Na^+^	ISFET	54 mV/dec	1·10^−5^–1·10^−1^ M	ISF3
Cl^−^	ISFET	−61 mV/dec	1·10^−5^–1·10^−1^ M	ISF4
${\text{NO}}_{3}^{-}$	ISFET	−59 mV/dec	6·10^−5^–2·10^−1^ M	ISF5
Ca^2+^	ISFET	29 mV/dec	6·10^−7^–1·10^−1^ M	ISF6

ORP and conductivity sensors both use chemically-inert Pt microelectrodes. The former measures redox potential between a working and a Dri-Ref reference electrode (World Precision Instruments, Sarasota, Florida, USA); the latter employs a 4-bar configuration, where an alternating current is applied between outer electrodes, and conductivity is measured between inner electrodes. ISFETs use a modified metal-oxide-semiconductor field-effect transistor (MOSFET) structure to obtain sensitivity to ion concentrations in an electrolyte. When placed in a solution, the channel conductivity of a MOSFET with the metal gate electrode omitted can be modulated by ion activity. Depositing an ion-selective polymeric membrane on top of the gate oxide and in contact with the electrolyte solution allows the sensor to measure concentrations of particular ions in combination with a (second) Dri-ref reference electrode. While the polymeric membranes are selective to one ion in particular, they do exhibit cross-responses to other ion types. Sensor fusion can then be applied to exploit such cross-sensitivity for higher performance.

As charge-sensing devices, ISFETs are susceptible to the buildup of residual charge, which can affect the measurement reading. While this drift effect may be tolerable and compensated within certain limits, excessive trapped charge may drive sensor biasing close to or beyond supply voltage levels. Either this effect or the sensitivity loss to changing ion activities due to aging of the sensing membranes may necessitate the replacement of the sensor. A temperature readout was also included because the sensors can be themselves influenced by temperature changes. Such variations may be encountered after transferring the sensor array through the air between beverage samples. By providing this information, the classifier is given the opportunity to compensate for—or otherwise exploit—the temperature dependence of the individual sensors.

Two readout custom boards connected the microsensors to the host system through a USB interface and handled sensor biasing, analog readout, and digitization. The six silicon microsensor channels were interfaced with a dedicated ISFET meter, while the other board provided integrated support for the conductivity, temperature, and ORP sensors (Giménez-Gómez et al., 2016).

#### 2.1.2. Intel Loihi Neuromorphic Processor

To demonstrate low-power edge tasting in real time, we trained our beverage classification models on the MicroBeTa dataset, and deployed them on one of the Intel Loihi neuromorphic chips (Davies et al., 2018) of the Kapoho Bay USB stick form factor (see Figure 1). Loihi is a digital multi-core processor optimized for running spiking neural networks. A single Loihi chip consists of 128 asynchronous neuro-cores with 1,024 current based (CUBA) Leaky Integrate and Fire (LIF) neurons each. Neural states and configurations are locally stored in the cores. Three synchronous ×86 processors are also included for handling input/output spikes and other general tasks such as monitoring and setting up the neural network.

### 2.2. The MicroBeTa Dataset

#### 2.2.1. Beverage Types

The beverage types selected for the MicroBeTa dataset are given in Table 2. This beverage selection covers a wide range of characteristics within a limited set of classes, with several semi-overlapping sets of attributes that could be expected to provide insight into how the data from various sensors could be used by the classifier: The red and white wine samples might be expected to be chemically similar due to sharing broadly analogous production processes, with the most obvious differences arising from complex organic chemicals rather than the simple measurements made by each separate sensor channel. The subgroup of still and sparkling water, red wine and cava covers four general cases arising from the presence or absence of carbonation and fermentation byproducts, respectively.

**Table 2:** Beverages represented in the MicroBeTa dataset, along with the label values they were assigned.

**Beverage**	**Commercial brand**	**Label**
White wine	Macabeu Celler Mas Bella 2017	0
Red wine	Merlot Duc de Foix 2015	1
Still water	Veri5	2
Sparkling water	San Pellegrino	3
Cava	Chardonnay Martí Serdà	4

The number of different beverage samples was limited by the time requirements and manual interaction necessary to obtain representative data from each additional beverage.

#### 2.2.2. Recordings

MicroBeTa comprises 7 h of temporal multivariate readings obtained from the electrochemical microsensor array described in section 2.1.1 when immersed in the various beverages (Table 2). The recordings were conducted at IMB-CNM during three sessions performed over the course of 3 days. Data from all sensors was read out continuously and simultaneously during each session, while the sensor array was moved from one beverage sample to another at fixed intervals of 5 min. During each transfer, the sensor array was washed with deionized water before being placed in the next sample to avoid unnecessary cross-contamination of subsequent beverages in the series.

The sequence of transitions between beverage samples was chosen to cover all combinations from one beverage to another. In this way, a dataset made up of complete measurement cycles—that is, cycles containing approximately 300 s of sensor readings from each beverage sample and following a predetermined sequence of transitions between beverages—remains balanced with respect to the number and types of beverages, the total and individual measurement time per beverage, and any effects due to contamination of the sensors with traces of the previous beverage. Yet several cycles from the second and third recording sessions were incomplete due to charge buildup or accidental contact between a sensor and the reference electrodes, needing the recording to be interrupted prematurely.

**Figure 2:**
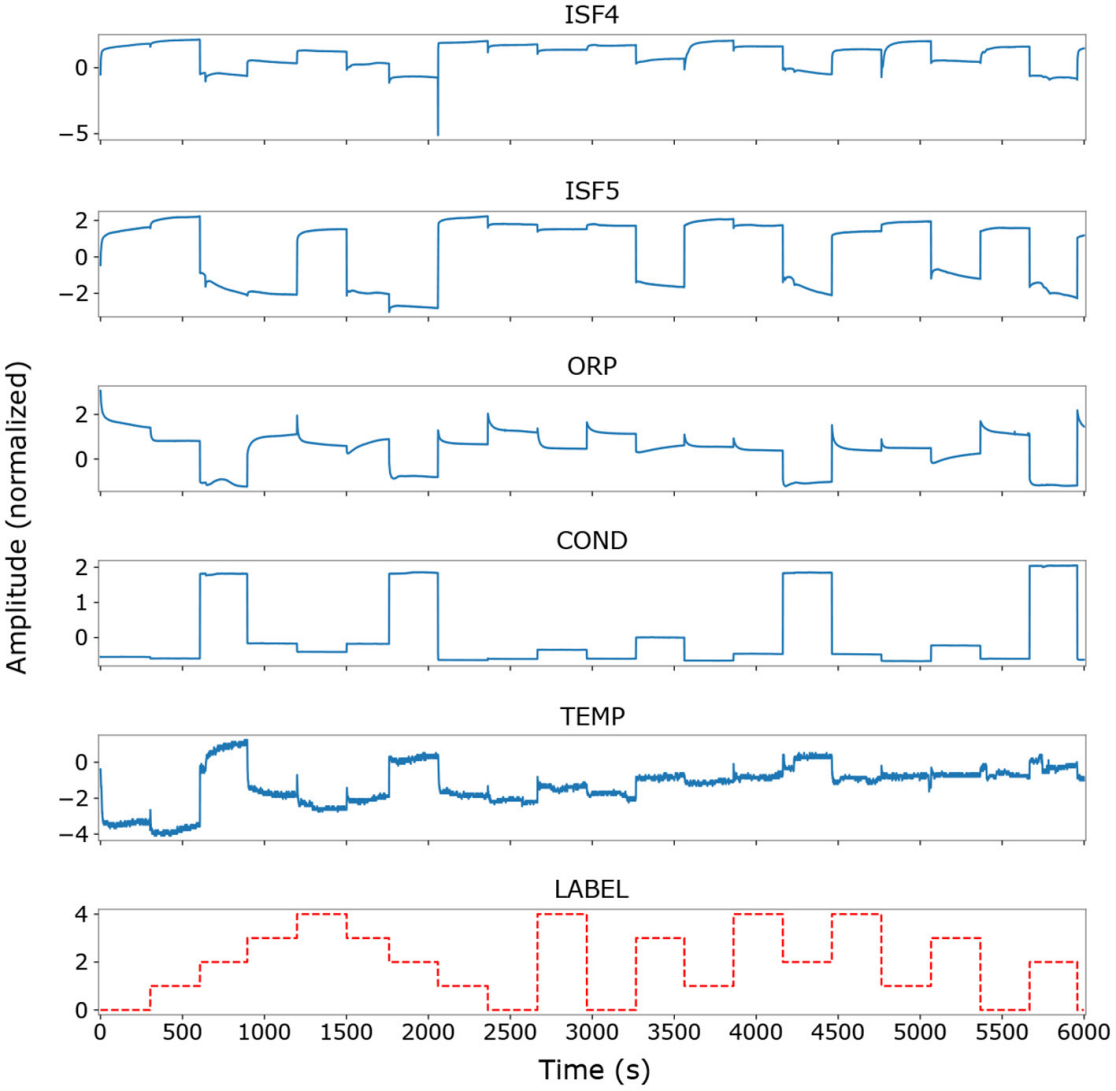
Raw time series data (Z-score-normalized) from the first recording session, showing traces from the Cl^−^, ${\text{NO}}_{3}^{-}$, redox potential, conductivity and temperature sensors over the first full measurement cycle. The remaining ISFET channels are omitted for clarity. Individual sensor signals and the associated labels are plotted against the sample index (corresponding to seconds of valid measurements). Note the predominance of constant-offset components over transient dynamics in the signals.

Data from each recording session was labeled manually, with the washing and transfer periods as well as transient instabilities of individual sensors assigned to a “non-beverage” class that was later discarded. Signals from all sensor channels were recorded at a rate of 1 Hz, resulting in approximately 26,000 labeled measurements across all three sessions. Figure 2 shows raw data from a complete measurement cycle of the first recording session used in this work. The number of measurements and data samples in relevant subsets of the data is listed in Table 3.

**Table 3:** Measurement (sensor readings) and sample (overlapping time windows of length 16) counts in the MicroBeTa dataset and relevant subsets thereof.

**Session**	**Raw measurements**	**Samples (window length 16)**
1st	11,931	11,673
2nd	8,535	5,355
3rd	5,579	4,169
Total	26,045	21,197

*Sample counts indicate the number of time windows available for learning, after preprocessing*.

### 2.3. Dataset Preparation

#### 2.3.1. Preprocessing

Several preprocessing steps were performed on the measurement data before it was used to train a classifier model. Incomplete measurement cycles in which not all beverages were recorded, or measurements of specific beverage samples that were much shorter than others, were removed entirely to preserve statistical balance with respect to both the total recording time per beverage and also the sequence of transitions between individual beverages. Any measurements lasting significantly longer than 5 minutes were truncated to that length.

The data from each recording session was filtered and normalized independently for several reasons. Not only were sensor offsets and sensitivities observed to change from one acquisition session to the next, but several sensors were replaced for the second (Na^+^, Cl^−^, Ca^2+^) and third (H^+^, Cl^−^) recording sessions due to performance degradation. Independent normalization additionally allowed to compensate for biases due to changes in ambient conditions between sessions. We removed any data points with corrupted beverage readings from one or more sensors, e.g., during transfer from one beverage to the next, sensor cleaning in deionized water, or accidental contact with reference electrodes.

**Figure 3:**
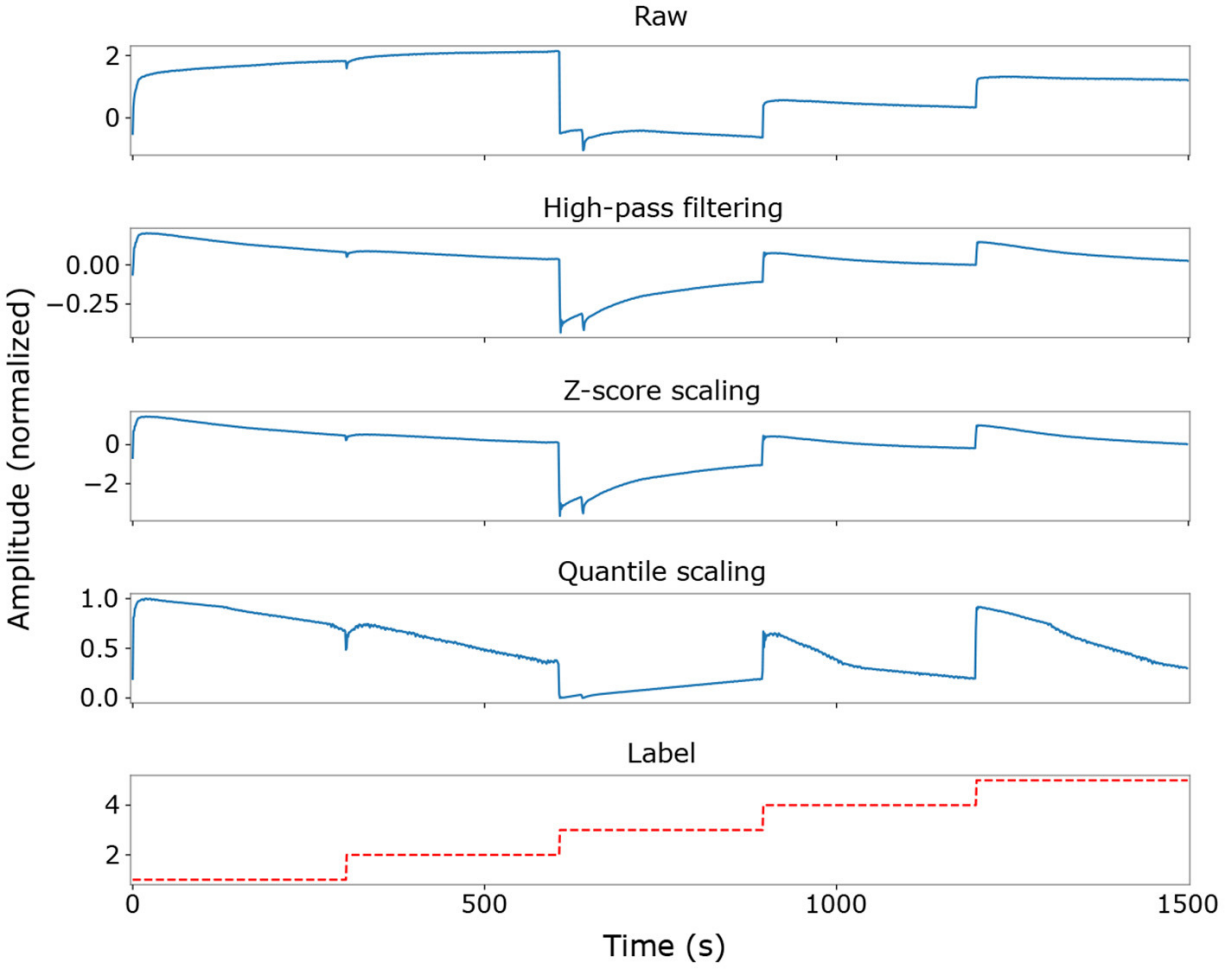
Dataset preprocessing transforms when using a high-pass filter with a cutoff frequency of 0.5 mHz, shown for the first 1,500 measurements in the dataset for a single ISFET sensor channel (Cl^−^). Note the nonlinear enhancement of near-zero signals by the quantile scaler. Signal traces are not continuous in time everywhere due to the removal of invalid measurements and edge discontinuities between labels.

The sensor readouts typically show large offsets corresponding to the various beverage types. The rate-based encoding scheme used when converting the trained classifier to a spiking network translates the constant offsets into dense spike trains, reducing the energy efficiency of the spiking model and masking the sparse dynamic signals which are more appropriate for neuromorphic processing. Encoding such high-magnitude offset components would also limit the range of signals that can be represented on low bit resolution systems commonly used in edge applications. Therefore, a configurable high-pass filter was used to attenuate level offsets in the input signals while emphasizing their dynamic components. Its transfer function was adapted to the MicroBeTa dataset by setting a pole at 0.5 mHz, a zero at the origin (i.e., at 0 Hz), and unity gain. In practice, values between 0.5 and 0.8 mHz were found to give accuracies higher than 90% in both ANNs and SNNs. Figure 3 illustrates the effects of the filter when applied to the dataset.

Outliers were removed following the filtering operation, and the resulting signal was subsequently normalized. Both operations were performed according to the statistics of training data only. Outlier values were deleted by excluding all measurements in which at least one sensor channel contained a value further than four standard deviations from the mean of that channel. Each sensor channel was then normalized independently using quantile normalization (Bolstad et al., 2003), which transforms the data to a normal distribution before nonlinearly mapping it to a uniform distribution on [0, 1]. Quantile-normalized data was found to preserve a high correlation between the ANN and the converted SNN, because the initial mapping to a normal distribution prevents a large fraction of input values from being pushed close to zero. Figure 3 shows the effects of this normalization method on the data.

#### 2.3.2. Sample Generation

Following normalization, the corresponding time series from each recording session were concatenated to produce a single, piecewise-normalized series for each sensor, from which samples could be drawn for training and validating a classifier model. The data samples used in this work are fixed-length time windows containing the signal values from all sensors over a contiguous range of timestamps.

The length of the time window may be chosen arbitrarily to correspond to a time scale of interest. To preserve causality, the label that corresponds to a given time window is defined as the label assigned to the latest measurement in the window. In this context, a sample is an array with shape *T* × *N*, where *T* is the length of a time window that slides over the multivariate time series, and *N* is the number of sensors. Therefore, the *i*-th labeled sample (*x*_*i*_, *l*_*i*_) in the dataset comprises the sample $x_{i}={⋃}_{k=1}^{N}[C_{k}(t_{i-T})\ldots C_{k}(t_{i})]$ and label *l*_*i*_ = *L*(*t*_*i*_) with *C*_*k*_ indicating the time series of the *k*-th channel (sensor) and *L* indicating the time series of labels. Note that the channel order is not relevant for the model architectures used in this work, and changing this order would not be expected to affect final performance.

**Figure 4:**
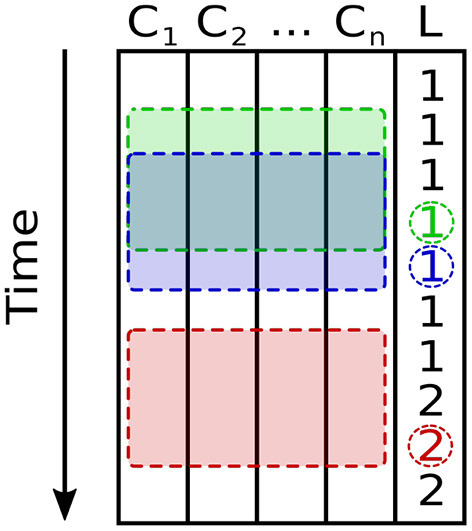
Scheme used for sampling a multivariate time series with channels *C*_1_…*C*_*n*_ for the *n* = 9 sensors, and label *L*. Two consecutive, overlapping samples (time windows) and their corresponding labels are shown in green and blue, respectively. A third time window, shown in red, would be discarded because the measurements it contains span multiple labels.

Because labels were available for every measurement, overlapping time windows were used to make the most of the available data. Time windows that contained measurements with multiple labels were discarded, as in these cases the beginning and end of the window contain measurements from different beverages with an implicit discontinuity between them. Therefore, two consecutive samples share all but the first and last values in each sensor time series. Figure 4 shows a diagram of the sampling scheme used in this work.

Shorter time windows are preferable to longer ones for several reasons: Firstly, the number of time windows discarded due to spanning several labels increases with the window's length, allowing a system using shorter time windows to make better use of a limited dataset. Secondly, they afford the trained system a shorter response time during online inference. Lastly, longer time windows require networks with a greater number of connections than would otherwise be necessary. The shortest sample length that suffices to capture the input signal's relevant dynamics should thus be preferred. A window length of 16 measurements (i.e., window length 16) was used throughout this work, corresponding to 16 s of sensor recordings.

#### 2.3.3. Other Practical Considerations

Most of the available samples from all recording sessions were used for training the ANN models, with the last complete measurement cycle from the final session reserved for testing. Our motivation not to sample the dataset randomly was twofold: On the one hand, the use of overlapping time windows means that if the dataset were sampled randomly, a large fraction of the measurements from each sample in the test set would be identical with measurements from several other samples in the training set; on the other hand, from the point of view of a given test sample, random sampling would allow the model to train on data samples subsequent to those used for test. This is not a condition that will happen in practice.

The recordings in the MicroBeTa dataset pose several potential challenges for a classification algorithm. In particular, three ISFET sensors had to be replaced before the second recording session; and two more sensors had to be replaced before the third session, as mentioned above. Furthermore, the first session contains significantly more data than either of the subsequent sessions after preprocessing, comprising 55% of the dataset as shown in Table 3. Because of the sequential sampling strategy and difference in session lengths, using all sessions means that the network is trained primarily on data from the first session, while the test set contains measurements from the third. Nonetheless, the models were ultimately found to generalize well. Such a good generalization could be favored by the small number of sensors replaced along the third session of recordings, and to the presence of full measurement cycles from that session in the training set.

### 2.4. Fusing Sensor Readings

#### 2.4.1. Neural Network Model

We aimed for a small, energy-efficient model size that fits in one of the two chips included in the Kapoho Bay platform, avoiding any overheads in terms of latency and power consumption that might be added in inter-chip communication.

**Figure 5:**
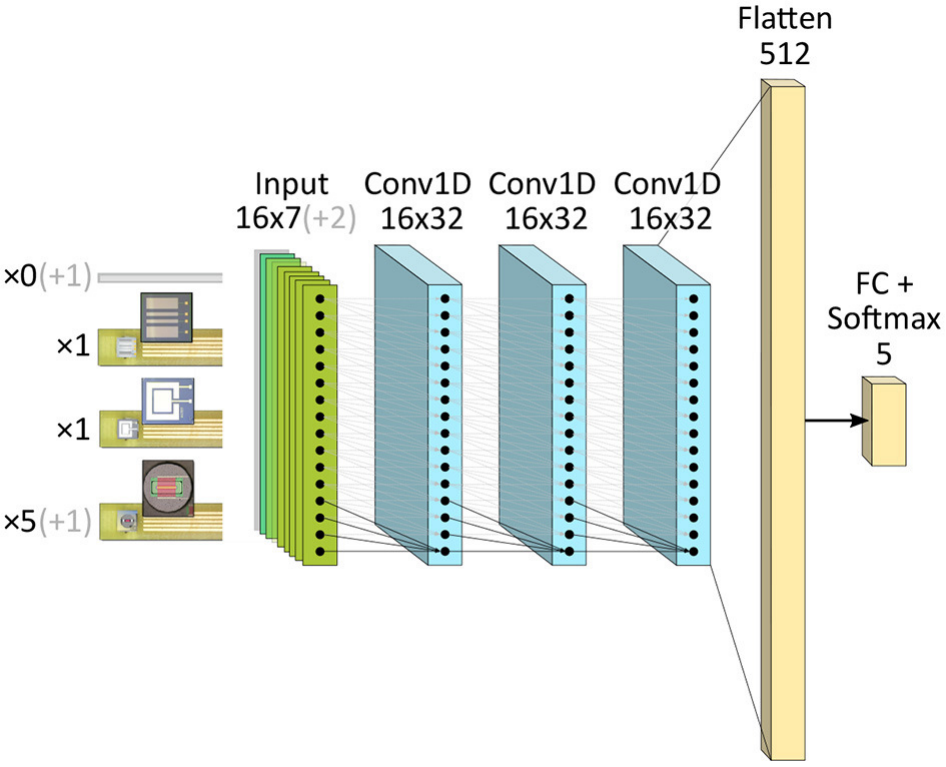
Causal CNN architectures implemented in the work. Opaque channels depict the final seven-sensor configuration deployed on all benchmarked systems. Transparent channels the additional sensor inputs originally used to classify the beverages from all nine sensors.

The results described below were obtained with networks consisting of three convolutional layers followed by a fully-connected Softmax classifier stage, where every sensor used corresponds to an input channel as depicted in Figure 5. We chose a deep convolutional architecture with the intent of incorporating future features such as learning new sensor inclusions or replacements in a continual manner. All convolutional layers in each model implement one-dimensional, causal convolutions along the time dimension of the input sample. A kernel size of four was used throughout, and each layer learned 32 convolutional kernels. No intermediate pooling operations were used, and appropriate padding ensured that the dimensions of the sample did not decrease as it passed through the network. Multi-layer classifiers were also explored to map the signal from the last convolutional layer to the target classes, but they were not found to provide sufficient improvement to offset their significant added complexity. All hyperparameters were swept through realizable ranges during training. No biases were used in the network as they can lead to reduced accuracy in the converted SNN unless carefully regularized during training (Rückauer et al., 2019).

While including a batch normalization layer following each convolutional step improved ANN performance and reduced training time significantly, it also increased variance in the SNN accuracy. For this reason, no normalization layers were used, and the training duration was increased from 200 to 1,500 epochs. Networks were trained with the AdaBound optimizer on categorical cross-entropy loss, with norm clipping and L2 regularization. A batch size of 128 samples was chosen.

#### 2.4.2. ANN-SNN Conversion and Mapping to Loihi

The classification models were initially trained in Keras and then converted to rate-based spiking networks using the SNN Toolbox (Rueckauer et al., 2017). The networks were then deployed on Loihi via the NxTF framework (Rueckauer et al., 2021), which provides a Keras-like API to instantiate neural networks on Loihi, and includes a compiler to optimally apply Loihi's resource sharing features while mapping convolutional topologies to the multi-core hardware.

The SNN Toolbox configuration used for the experiments in this paper was made available together with the dataset. Sensor readings were mapped as bias currents of the input SNN layer, and membrane potentials were reset by subtraction via 2-compartment neurons after spiking. The weights of the neural network were normalized setting the maximum ReLU activations in each layer to the 99-th percentile of their total activity distribution. Finally, the output softmax layer was converted to spiking by computing spike rates according to the membrane potentials of the output neurons (Rueckauer et al., 2017). Keeping the CNN depth to three layers helped mitigating the accumulation of discretization errors across the hierarchy, an effect deeper rate-based converted SNNs are more prone to. Each of the test samples was ran for 50 algorithmic time steps on the Loihi (Davies et al., 2018).

#### 2.4.3. Selecting Informative Sensors

We determined the contribution of each sensor to beverage classification via the random forest (RF) (Breiman, 2001) algorithm with Gini impurity. RF is an efficient dimension reduction method that uses a simple bagging ensemble technique. RF models comprise many decision trees, and each decision tree learns how to split the input into smaller subsets to predict the beverage class. Sensor importance is assessed by looking at how much each sensor contributes to each tree, then taking an average value over all the decision trees, and finally comparing the contributions of each sensor. The Gini impurity is the default metric used in the scikit-learn toolbox to determine how the trees are split at the different nodes. For a decision forest (in this work, we used 1,000 trees), it is possible to calculate the average reduction in the impurity of each sensor and use the average reduction as the criterion for sensor selection.

### 2.5. Benchmarking Accuracy and Energy Efficiency

ANN and SNN performance statistics were obtained from five models per ANN input configuration, independently trained varying only the initialization seed value. All other hyperparameters were held constant for all experiments. As a baseline, we studied the accuracy of the k-NN algorithm over a sweep from 1 to $2\sqrt{N}$ nearest neighbors, where *N* is equal to the 19,844 training samples of the dataset.

We estimated energy efficiency from the dynamic power consumption and the inference time measured when running the SNN on one of the Kapoho Bay's Loihi chips. Dynamic power was obtained as the additional cost of performing inference with respect to the baseline idle power, i.e., when the device is powered and clocked, but idling. Its relative performance in these same terms was compared with the results obtained from ANNs deployed on two other devices for DNN acceleration: an Nvidia GeForce GTX TITAN X GPU, and an Intel Movidius Neural Compute Stick 2 (NCS2) for low power edge-AI inference. A batch size of 1 was used for all power measurements.

Power consumption on the NCS2 was monitored by means of an external UM34C USB power meter. In order to account for I/O power consumption, we evaluated an upper bound of the I/O power by running a single-layer softmax perceptron with the same number of inputs and outputs, and by subtracting inference power from the power consumption measured after allocating the models on the device. This upper bound of I/O power was then deducted from the dynamic power exhibited by the NCS2 when running the neural network models.

## 3. Results

### 3.1. Classification Accuracies vs. Sensor Selection

We evaluated both k-NN and CNN accuracies initially using all nine sensor readings from the test dataset. The first row of Table 4 shows the accuracies reached for this sensor combination using a rolling window of 16 readings. Accuracy values refer to the per-class accuracy, averaged over all time windows of the test set. The ANN outperforms the top scoring k-NN (i.e., a 201-NN) by a 3%. Compared to the ANN, the SNN accuracy dropped approximately 1% on average, with similar standard deviation.

**Table 4:** Average classification performance of ANN and converted SNN models on the MicroBeTa dataset.

**Dataset**	**Test accuracy**		
	**k-NN**	**ANN**	**SNN**
9 Sensors	88.8%	91.3 ± 0.9%	90.7 ± 0.8%
7 Sensors	98.2%	98.6 ± 0.6%	97.0 ± 1.6%

*Statistics were obtained by training each configuration independently five times and varying only the initialization seed value. All other hyperparameters were held constant for all experiments. Error intervals are given as the standard deviation*.

We also tested the performance of every sensor channel on the k-NN algorithm. In all cases, the accuracy of a single sensor does not exceed 63% (see Table 5). When fusing the outputs of each readout board via the k-NN method, using ISFETs alone (93.8%) increases the accuracy by 5% compared to the accuracy (88.8%) from using all the sensors of the array. Combining conductivity, ORP, and temperature sensors gives 85.6% accuracy. The high accuracy of using only ISFETS could be due to the fact that the training set already contained data from all the replaced sensors and the day-to-day drifts that were present in the test data, thus obviating the need for more stable sensors to compensate for these effects.

**Table 5:** Individual accuracies reached by a k-NN classifier when using single sensor channels.

**Sensor type**	**TEMP**	**ISF1**	**ISF2**	**ISF3**	**ISF4**	**ISF5**	**ISF6**	**COND**	**ORP**
Accuracy	21.4%	53.5%	35.6%	57.2%	61.6%	38.0%	34.0%	58.5%	62.7%

To validate the effectiveness of choosing the most informative sensors, we computed the importance value of the nine sensors using the RF algorithm. The results in Table 6 show that two of the sensors (TEMP and ISF5) are least informative. All models were retrained for the seven sensor combination, achieving the results shown in the second row of Table 4. The k-NN (a 1-NN in this case), ANN and SNNs are on average 11, 8, and 7% more accurate, respectively, than the nine-sensor configurations. Selecting the seven most informative sensors helped boosting performance in these terms.

**Table 6:** Sensor importance values estimated using the random forest algorithm.

**Sensor Type**	**TEMP**	**ISF1**	**ISF2**	**ISF3**	**ISF4**	**ISF5**	**ISF6**	**COND**	**ORP**
Importance value	0.025	0.092	0.176	0.126	0.124	0.044	0.119	0.185	0.109

*TEMP and ISF5 channels in particular contribute little information*.

**Figure 6:**
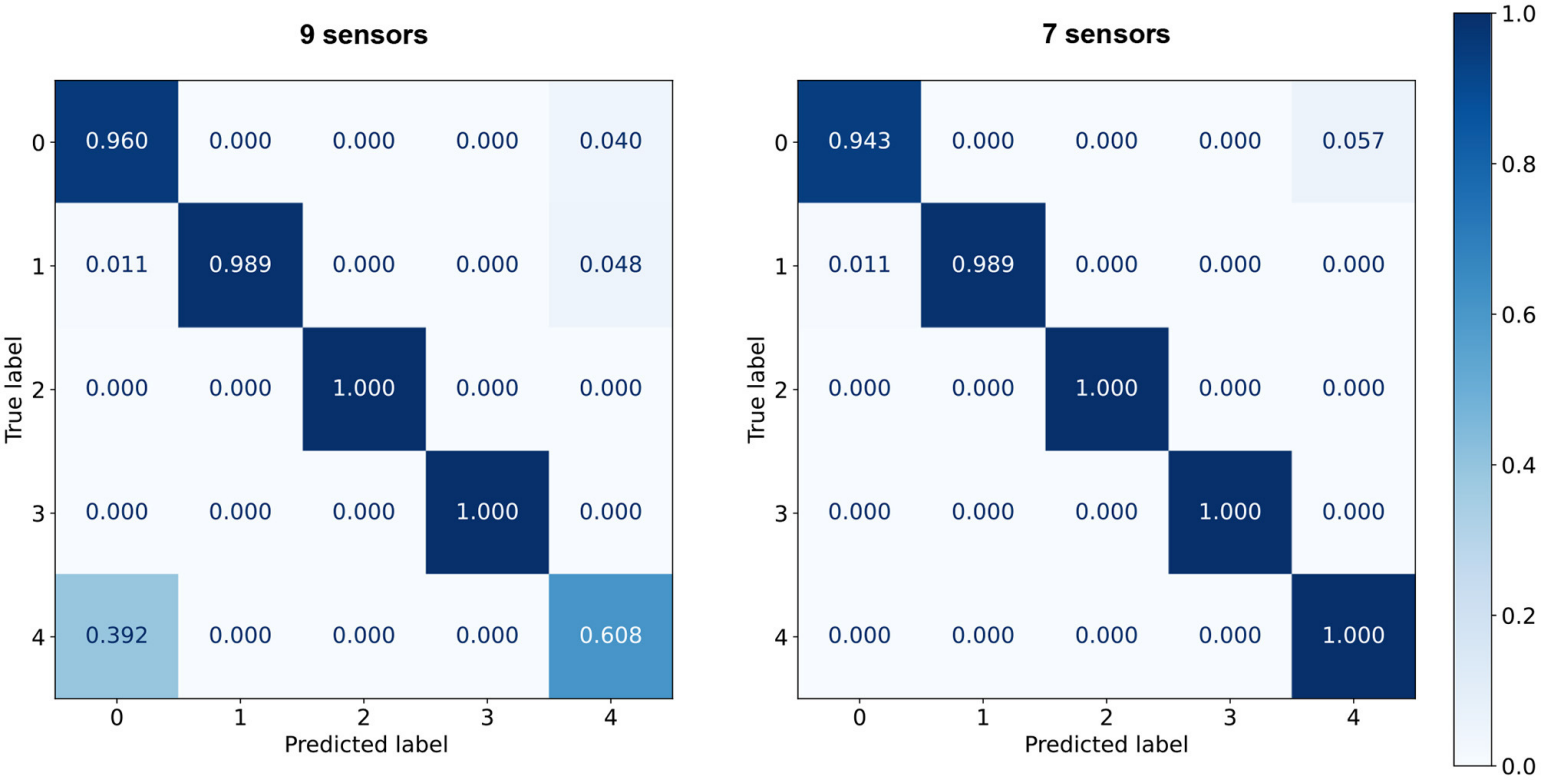
Confusion matrices summarizing the CNN prediction results for the nine **(left)** and seven **(right)** sensor combinations. Classification performance is averaged over five independent runs of CNNs trained with random initialization seeds.

For the selected sensor combination, the ANN still exhibits higher accuracy than the 98.2% achieved by the 1-NN. It also surpasses the 97.5% accuracy of a perceptron similar to the model used to estimate I/O power on the Movidius. When training the CNN, the variability of SNN accuracies was found to worsen considerably with lower model complexities. The models tended to overfit when increasing the number of kernels beyond the chosen value of 32. Besides its suitability for fusing data from multiple channels with a reduced number of weights, the small convolutional architecture of Figure 5 was chosen for two more reasons: for its translational invariance, which would help to pick up relevant input features even in dynamically changing sensor readings; and for having a low but enough dynamic energy consumption to allow external metering of inference power, distinguishable from I/O power on the Movidius. Figure 6 shows confusion matrices of test accuracies averaged over the five final CNN model results.

### 3.2. Time Delay Response

**Figure 7:**
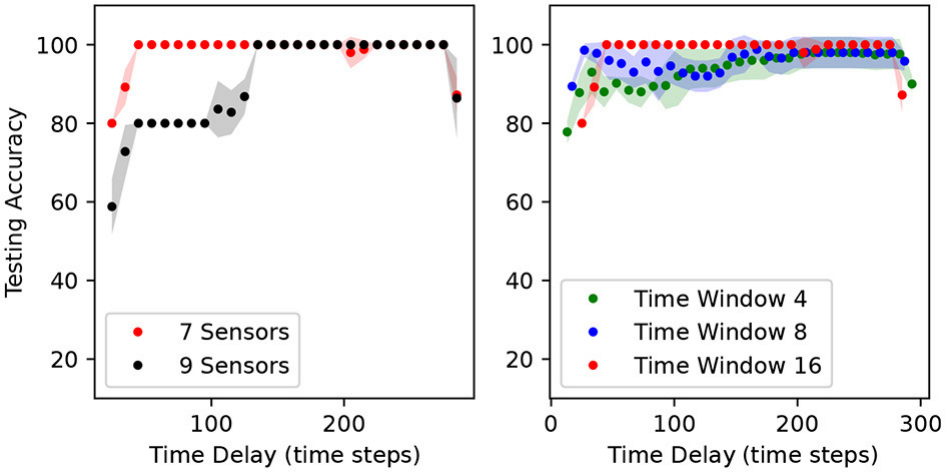
Classification accuracy vs. time delay for the seven and nine sensor combinations **(left)** and accuracy for the seven sensor combination for different time window lengths **(right)**. The dots indicate the mean and the shadowed regions indicate the standard deviation over five runs of ANNs independently trained with random initialization seeds.

We computed the online accuracy classification vs. time delay fixing window length to 16 for the two sensor combinations. The results are shown in Figure 7. A prediction is done at every time step. The dots indicate the average accuracy over 10 time steps. The accuracy varies between 80 and 100% for the seven-sensor combination and between 58.8 and 100% for the nine-sensor combination throughout this period of 10 s. The online accuracy using seven sensors is higher than using nine sensors at almost every time step.

The length of the time window is important because it affects the accuracy and the response latency. We investigated how the online accuracy of a network trained on the seven-sensor combination changed with the time window length. From the results in Figure 7, it is clear that a larger time window leads to a higher network accuracy but also results in a longer latency response. The choice of the window length is thus dependent on the desired accuracy-latency trade-off.

### 3.3. Dynamic Energy Consumption

Table 7 compares dynamic power, inference time, and dynamic energy per inference across hardware devices for the selected seven-sensor combination. Our neuromorphic implementation of the CNN for artificial taste consumes 15 mW of dynamic power on the Kapoho Bay, 49× and 643× lower than the same model running as non-spiking ANN on the NCS2 and the GPU, respectively. Power efficiency came at the cost of increasing the inference time to allow the spiking rates to converge; our SNN runs almost 2× and 3× slower on the Loihi than the ANNs deployed on the other two devices. Notably, the SNN still achieves remarkable gains in terms of dynamic energy cost—15× lower than the NCS2 and 290× lower than the GPU—requiring only 0.1 mJ per inference. This value is one order of magnitude lower than the dynamic energy reported on the k-NN implementations of Frady et al. (2020), when processing 76,800-sample datasets on Loihi.

**Table 7:** Average dynamic power consumption, inference time, and dynamic energy cost per inference across hardware devices when using a combination of seven sensors.

**Hardware**	**Dyn. power**	**Inf. time**	**Dyn. energy**
	**(mW)**	**(ms)**	**(mJ/Inf)**
GPU (Titan X)	9,649	3.0	29.0
Movidius (NCS2)	736	2.0	1.5
Loihi (KB)	15	5.6	0.1

## 4. Discussion

This work introduces the first neuromorphic implementation of artificial taste. Our electronic tongue uses rate-based, deep spiking convolutional networks to fuse dynamic, electrochemical microsensor readings. It performs with high accuracy (97%) and high energy efficiency (0.1 mJ per inference), and it can run the neural networks in real time (5.6 ms, over 178× lower than the sampling period of the sensor readout employed by the system) on a single Loihi chip. The system exploits sparse spiking computation and the particular latency budget of chemical sensor dynamics to trade off inference time for power consumption, achieving energy efficiency gains of 15× and 290× when compared to the same CNN architecture running with continuous values on the low-power Intel Movidius Neural Compute Stick 2, and on an Nvidia GeForce GTX TITAN X GPU, respectively. According to Frady et al. (2020), the proposed CNN would be an order of magnitude more energy efficient than the k-NN when classifying from 76,800 training examples on the Loihi; the approximate k-NN algorithm would distribute the training set among multiple chips. In contrast to the convolutional network, both k-NN power consumption and chip occupancy would scale up substantially with the addition of more temporal samples in the dataset. Furthermore, the CNN is better suited for extensions such as learning online from dynamically changing data due to e.g., sensor replacements.

Neuromorphic computing is advantageous in electrochemical monitoring, as sensors follow slow dynamics that relax inference speed requirements. This allows for the 2× -3× increase in latency needed by the SNNs to reach accuracies comparable to the ANNs without noticeable lags. Contrary to previous implementations of electronic tongues found in literature, our system is capable of classifying solutions accurately and continuously over transient sensor responses, with no need to delay inference until steady state is reached. This characteristic may be crucial in preventing critical risks (e.g., product contamination, process malfunction) early, when incidence is still low.

Domain-optimized CNN architectures for sequential data offer efficient training and representation of time-invariant features in a conceptually and computationally straightforward manner. Furthermore, this class of architectures is well understood and widely supported, affording easier integration with existing systems, and allowing more direct translation into the physically-constrained synaptic connections of neuromorphic hardware. While Recurrent Neural Network (RNN) models can be more efficient on such hardware by operating on instantaneous measurements—instead of on sequential values of the data stored with fixed-time window lengths—it still remains challenging to train and achieve the same levels of accuracy with spiking recurrent architectures.

To evaluate system performance (Davies, 2019), we created MicroBeTa: a beverage tasting, benchmark dataset to discriminate between five commercial beverage varieties using temporal readings acquired from a solid-state electrochemical microsensor array. The dataset covers every combination of transitions from one beverage to another, including all water, wine, and cava types in each arrangement to balance the set. It extends to 26 ksamples (about 7 h) of dynamic multivariate data, including sensor drifts and replacements. As the first open dataset of its kind, we believe that it will be useful for the research community to explore and compare different spike encoding or processing approaches applied to this new sensory domain. This benchmark can be used in a real-time, non-batched regime as a preliminary workload to assess the latency, throughput, accuracy, energy, or resource consumption of alternative neuromorphic implementations for chemical monitoring applications.

Our studies show that accuracy can be improved by fusing readings from all input channels except for the temperature and ${\text{NO}}_{3}^{-}$ ISFET sensors. Excluding these last two sensors makes sense given the negligible thermal effects on sensor readings, and the erratic response exhibited by FET sensor in some measurement cycles. These results corroborate the complementarity of all the remaining microsensors of the array toward generating a unique fingerprint for each beverage of the dataset (Legin et al., 1999).

When miniaturizing an autonomous electrochemical monitoring system, power consumption becomes one of the main technical challenges limiting the inclusion of embedded intelligence. Our spiking taste implementation curtails inference costs to an average of 100 μW at the 1-Hz multisensor sampling frequency used in this work. This power budget is close to that of state-of-the-art smart electrochemical sensing devices, which integrate the chemical sensors and the CMOS readout circuitry with a power consumption of tens of μW per sensor (Li et al., 2017; Miscourides and Georgiou, 2019). For the number of sensors selected in the work, power requirements could be satisfied up to several months by a single CR2032 coin cell. Such an energy autonomy opens the door for deploying chemosensory integration directly on the edge, avoiding the communication bottlenecks, delays, and privacy concerns inherent to cloud computing. The results also indicate good robustness to sensor non-idealities, using dynamic readings in an architecture manufacturable at wafer level. Once the functionality of our neuromorphic tasting approach is verified, the SNN could be applied to continuous monitoring in remote and compact locations, providing an appropriate environment to study incremental learning of new classes or sensor channels.

## Data Availability Statement

The dataset introduced in this study can be found in the Zenodo open access repository, with link http://doi.org/10.5281/zenodo.5457501.

## Author Contributions

NL and BR contributed to the simulation work, energy measurements on hardware platforms, and writing. PS contributed to the simulation work and writing. MR and CJ-J contributed to the sensor fabrication and data collection. S-CL contributed to the experiment conception and writing. JM-T contributed to the experiment conception, data collection, simulation work, energy measurements on hardware platforms, and writing. All authors contributed to the article and approved the submitted version.

## Funding

This work was supported by the European Union's Horizon 2020 research and innovation programme under the Marie Sklodowska-Curie grant agreement no. 747848 and the SNSF-Sinergia WeCare project (N°CRSII5_177255).

## Conflict of Interest

The authors declare that the research was conducted in the absence of any commercial or financial relationships that could be construed as a potential conflict of interest.

## Publisher's Note

All claims expressed in this article are solely those of the authors and do not necessarily represent those of their affiliated organizations, or those of the publisher, the editors and the reviewers. Any product that may be evaluated in this article, or claim that may be made by its manufacturer, is not guaranteed or endorsed by the publisher.
